# Stepping away from the scroll: a chain mediation model from physical exercise and adolescent short video addiction

**DOI:** 10.1186/s40359-025-03710-z

**Published:** 2025-12-22

**Authors:** Bin Peng, Xingtao Zhu, Tong Han, Subing Zhao, Jiayi Zheng, Fengshuo Jia

**Affiliations:** 1 Nanfang College Guangzhou, Guangzhou, China; 2 Gdansk University of Physical Education and Sport, Gdansk, Poland; 3 School of Physical Education, Shihezi University, Shihezi, China; 4 Shanxi Finance & Taxation College, Taiyuan, China

**Keywords:** Physical exercise, Psychological resilience, Depression, Short video addiction, Adolescents

## Abstract

**Objective:**

In this study, the relationships among physical exercise, psychological resilience, depression, and short video addiction were examined, and the sequential mediating roles of psychological resilience and depression were tested, with the objective of providing theoretical support for adolescent mental health interventions.

**Methods:**

In September 2024, convenience sampling was used to recruit 1,002 adolescents from 6 primary and secondary schools in Hunan, Sichuan, Shaanxi, Shanxi, and Anhui Provinces, China. Paper questionnaires were administered and collected in classrooms by researchers and teachers. The survey covered sociodemographic information and four standardized scales: the Physical Activity Rating Scale, the Patient Health Questionnaire Depression Scale (2 items), the simplified Connor–Davidson Resilience Scale, and the Short Video Addiction Scale. Data were analyzed with SPSS 23.0 for descriptive and correlational statistics, and sequential mediation was tested using the PROCESS macro (Model 6).

**Results:**

Physical exercise was negatively correlated with depression (r = -0.260) and short video addiction (r = -0.269) and positively correlated with psychological resilience (r = 0.229). Psychological resilience was negatively related to depression (r = -0.174) and short video addiction (r = -0.186), whereas depression was positively related to short video addiction (r = 0.345). Mediation analysis confirmed that psychological resilience and depression sequentially mediated the relationship between physical exercise and short video addiction.

**Conclusion:**

Physical exercise influences short video addiction both directly and indirectly by enhancing psychological resilience and reducing depression. These findings highlight the protective role of physical exercise in mitigating short video addiction and provide theoretical evidence for adolescent mental health interventions.

## Introduction

Adolescence is a critical stage in human development and is marked by rapid physical growth and significant psychological changes. Physical exercise (PE) not only promotes physical growth but also profoundly affects mental health, cognitive ability, and social adaptability [1]. As awareness of health issues increases, more individuals recognize the benefits of PE, especially its positive role in disease prevention and enhancing quality of life. PE refers to systematic physical activities that are aimed at improving strength, endurance, flexibility, and coordination, thus promoting both physical and mental health. It encompasses various forms, such as aerobic exercise, strength training, flexibility exercises, and balance training, all intended to improve fitness, enhance one’s physical condition, and effectively prevent or alleviate health problems [2]. A 2022 meta-analysis of 13,884 studies on adolescent physical exercise compared sedentary and active students and revealed that regular participation in exercise plays a crucial role in enhancing fitness levels and body composition among youth aged 12–16 [3]. Thus, a lack of PE in adolescents can lead to a weakened immune system [4], increased depression and anxiety [5], decreased quality of life [6], and increased weight and obesity [7]. Research on the role of PE in adolescents is not only relevant to individual health but also to academic performance and future development, and it provides scientific evidence for optimizing educational systems and public health policies. Exploring the mechanisms through which PE affects health will aid in the development of precise interventions to enhance adolescents’ overall well-being and contribute to the promotion of public health.

Short video addiction (SVA) refers to a behavioral dependency marked by an overreliance on short video platforms. Individuals with this addiction display persistent or frequent short video watching habits and struggle to control their viewing time. Despite being aware of negative impacts such as reduced academic performance, social isolation, and sleep deprivation, individuals find it challenging to regulate or reduce their screen time [8]. Taking TikTok as an example, this globally popular short video platform has amassed more than 1.677 billion users, with 1.1 billion active users spread across more than 160 countries and regions [9]. The massive size of the user base not only demonstrates its global influence but also highlights the profound impact of short video platforms on lifestyles and social interactions in the digital age. According to the latest short video development report in China (2024), the number of short video users in China has reached 1.05 billion, accounting for 95.5% of total internet users, with the proportion of adolescent users steadily increasing [10]. Prolonged engagement with these platforms may lead to decreased cognitive abilities, attention deficits, and impaired information processing [11]. Therefore, investigating SVA among adolescents helps in understanding its behavioral patterns and psychological mechanisms, providing a scientific basis for effective intervention strategies. According to self-regulation theory [12], PE can enhance an individual’s self-regulation abilities, improving self-control and promoting delayed gratification. Short video addicts often demonstrate poor self-regulation and struggle to control the excessive use of such platforms. PE, which requires persistence and discipline, can improve executive functions and self-control, reducing dependency on short videos [13]. Therefore, PE can influence adolescent SVA. Earlier research has consistently reported an inverse association between PE and SVA. For instance, a cross-sectional survey of 756 adolescents in Chongqing and Chengdu, China, revealed that PE reduced SVA, with self-control acting as a mediator. Moreover, cumulative ecological risk was found to moderate this relationship [14]. Hence, PE plays a crucial role in reducing the risk of SVA. On the basis of these findings, this study hypothesizes a negative relationship between PE and SVA (H1).

In today’s rapidly changing social environment, adolescents are confronted with multiple pressures and challenges. Psychological resilience (PR), which is regarded as a key psychological trait that supports adaptation and recovery in adverse circumstances, has become a central topic in mental health research [15]. It describes the ability to maintain stability, adapt positively, and even achieve growth when facing stress, setbacks, or major difficulties [16]. From the perspective of psychological capital theory [17], resilience—together with optimism, self-efficacy, and hope—constitutes an important psychological resource that enables individuals to manage stressors effectively. Consequently, PE contributes not only to physical health but also to adolescents’ capacity to handle psychological pressure, serving as an important avenue for fostering resilience. Empirical evidence has demonstrated this effect; for instance, a cross-sectional survey of 1,613 adolescents across 15 Chinese provinces indicated that PE enhanced resilience, which subsequently improved self-efficacy [15]; thus, PE not only promotes physical health but also subtly enhances an individual’s ability to cope with stress and adversity, providing an important path for improving adolescent PR. Previous studies have shown that PE enhances adolescents’ PR. For instance, a cross-sectional study involving 1,613 adolescents from 15 provinces in China revealed that PE increased PR, which in turn improved self-efficacy [18]. Furthermore, from the perspective of self-control theory [19], PR is closely tied to self-regulation. Adolescents with higher levels of resilience generally display stronger impulse control and delayed gratification, enabling them to resist the appeal of short video content and thereby reducing the risk of problematic use. In contrast, those with lower levels of resilience may rely excessively on short videos to relieve negative emotions, increasing the likelihood of dependence or even addiction [20]. Thus, resilience functions not only as an adaptive capacity but also as a psychological protective factor against SVA. In support of this, a study with 560 participants revealed that higher levels of resilience predicted lower rates of SVA [21]. On this basis, the present research proposes that resilience mediates the pathway between PE and SVA among adolescents (H2).

Depression (DPE) is a complex psychological disorder that not only affects emotional experiences but also significantly impairs cognitive functions and social adaptability [22]. According to a 2021 meta-analysis synthesizing 72 studies conducted between 2001 and 2020, the global prevalence of depressive symptoms among adolescents was estimated to be approximately 37% [23]. Another 2019 meta-analysis of Chinese adolescents, which included 18 studies with 29,626 participants, revealed a 19.85% prevalence of depressive symptoms [24]. According to cognitive–behavioral theory [25], PE can alleviate depressive symptoms by changing individuals’ negative cognitive and behavioral patterns. Exercise enhances attention control and self-regulation, enabling individuals to better cope with stress and negative emotions. As a positive activity, PE can replace negative coping mechanisms (e.g., excessive short video use and social avoidance), thus reducing depressive symptoms. Therefore, PE influences adolescent DPE. Previous research has shown that interventions involving PE can reduce depressive symptoms among adolescents. For example, a retrospective review of 2,435 studies in 2025, including 23,681 healthy adolescents, revealed that PE interventions significantly reduced DPE among adolescents [13]. Hence, there is a significant correlation between PE and DPE. Additionally, according to the theory of self-regulation deficits [26], depressive emotions may impair an individual’s self-regulation abilities, making it difficult to control short video usage and increasing the addiction risk. Depressed individuals often experience impaired executive functions, such as reduced impulse control, attention deficits, and poor self-discipline [27]. The fragmented and highly stimulating nature of short videos makes them among the most addictive forms of media for individuals with DPE. Owing to the immediacy and habitual nature of short video usage, depressed individuals often struggle to set clear limits on their viewing time, leading to prolonged overuse [20]. Previous studies have shown that DPE is associated with addiction behaviors. For example, a cross-sectional study involving 525 Chinese adolescents revealed that depressive symptoms were positively correlated with mobile phone addiction behaviors [28]. Therefore, DPE is closely related to SVA among adolescents. On the basis of these findings, this study hypothesizes that DPE mediates the relationship between PE and SVA (H3).

In recent years, the increasing prevalence of mental health issues among adolescents has led to growth in studies seeking to identify the key factors that influence their psychological well-being. Given that adolescence represents a critical developmental stage for psychological maturation and emotional regulation, investigating the relationship between PR and DPE holds substantial theoretical and practical significance for fostering mental health in this population [29]. Bronfenbrenner’s ecological systems theory posits that PR is not merely an individual trait but is shaped by the interplay of multiple environmental systems, including family, school, and the broader social context. Similarly, the dynamic adaptation model conceptualizes resilience as an individual’s capacity to positively adapt to stress through continuous interactions with various ecological systems. This adaptive capacity plays a crucial role in mitigating the effects of persistent or recurring negative emotional states, such as DPE [30]. Empirical evidence supports the role of resilience in enhancing adolescents’ emotional regulation. For example, a large-scale cross-sectional study involving 6,401 adolescents aged 9 to 15 years revealed that higher levels of PR significantly attenuated the detrimental effects of depressive symptoms, thereby contributing to improved overall well-being [31]. These findings underscore the importance of resilience as a protective psychological resource for alleviating depressive symptoms and promoting mental health among adolescents (H4).

**Fig. 1:**
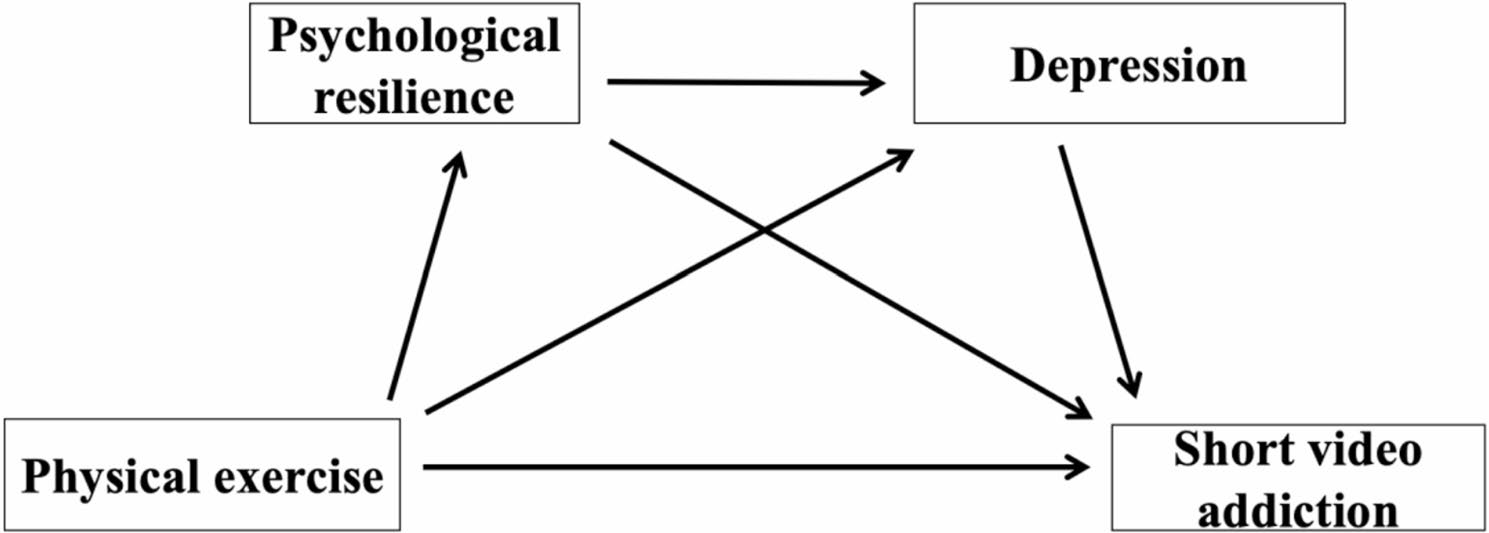
Hypothetical mediation model

Building on the aforementioned theoretical frameworks and empirical findings, physical activity has been consistently shown to be negatively associated with SVA. Moreover, PR and DPE may function as key mediating mechanisms underlying this relationship. Specifically, engagement in physical activity is believed to increase individuals’ levels of PR, thereby improving their capacity to cope with stress and regulate negative emotions. This enhanced resilience may in turn reduce the likelihood of experiencing depressive symptoms. Furthermore, lower levels of DPE are associated with a decreased tendency toward SVA, as individuals may be less inclined to use digital media as a maladaptive coping strategy. Accordingly, the present study proposes a chain mediation model in which PR and DPE sequentially mediate the relationship between physical activity and SVA (see Fig. 1). This model aims to elucidate the underlying psychological pathways through which physical activity may mitigate digital addictive behaviors among adolescents.

## Methods

### Participants

In Sept. 2024, a convenience sampling method was used to recruit 1,098 adolescents from six primary and secondary schools in Hunan, Sichuan, Shaanxi, Shanxi, and Anhui provinces, China. Prior to the survey, written informed consent was obtained from parents or legal guardians, and the study had been approved by the Ethics Committee of the authors’ institution. The questionnaire was administered in paper form and distributed as well as collected in classrooms by trained researchers and class teachers before the test. The first page of the questionnaire provided information about the study and the informed consent form; participants were required to sign “Agree” after reading to proceed with the survey. Only fully completed questionnaires were included in the analysis. Students who declined to participate or withdrew during the process had their right to withdraw fully respected and were not affected in any way. The first page of the questionnaire also specified the study purpose, survey content, data anonymity, confidentiality, and intended use. Participants typically completed the survey within about 10 min. Data screening was performed on the returned questionnaires, and those with excessively short response times or evident patterned responses were excluded. A total of 1,002 valid questionnaires were finally obtained. The study included 476 male participants (47.5%) and 526 female participants (52.5%), with a mean age of 14.78 years (SD = 1.589). The distribution by grade level was as follows: 35 primary school students (3.5%), 394 junior high school students (39.3%), and 573 senior high school students (57.2%). Regarding place of residence, 719 students (71.8%) were from urban areas, and 283 students (28.2%) were from rural areas. In terms of family structure, 223 students (22.3%) were only children, while 779 students (77.7%) were not. With respect to boarding status, 320 students (31.9%) were boarders, and 682 students (68.1%) were day students. Additionally, 499 students (49.8%) were left-behind children, whereas 503 students (50.2%) were not.

### Measurement instruments

#### Physical exercise

PE was assessed using the PE Rating Scale revised by Liang Deqing et al. [32], which evaluates three dimensions—intensity, frequency, and duration—each represented by one item. Responses were rated on a 5-point Likert scale. The overall score was computed using the formula: Exercise Score = Exercise Intensity × (Exercise Duration − 1) × Exercise Frequency. The resulting total ranged from 0 to 100, with higher values reflecting greater engagement in physical activity. In this study, the scale demonstrated acceptable internal consistency (Cronbach’s α = 0.669).

#### Depression

Depressive symptoms were assessed with the two-item Patient Health Questionnaire (PHQ-2) [33], a validated screening tool for adolescents. Participants rated two items on a 4-point Likert scale (1 = not at all, 4 = nearly every day) reflecting symptom frequency over the past two weeks. Scores range from 2 to 8, with higher values indicating greater severity. In this study, the PHQ-2 showed acceptable internal consistency (Cronbach’s α = 0.742).

#### Psychological resilience

PR among adolescents was measured with a short form of the Connor-Davidson Resilience Scale (CD-RISC) [34], originally developed as a 10-item instrument widely employed across diverse cultural contexts to evaluate individual resilience. Previous research has highlighted specific items within the scale as being particularly representative of core resilience dimensions. For example, items 1 and 5 have been identified as central indicators of resilience in several studies [35–37], while Waddimba et al. [35], through item response theory analysis based on a U.S. sample, found that items 2 and 9 demonstrated higher reliability and were more suitable for constructing a brief version of the scale. Guided by these findings and considering the characteristics of the present sample, the current study adopted items 2 and 9 to assess PR. Responses were scored on a 5-point Likert scale ranging from 1 (never) to 5 (always), with total scores ranging from 2 to 10. with total scores ranging from 2 to 10. Higher scores reflected stronger PR. In this study, the two-item version showed acceptable reliability (Cronbach’s α = 0.669), supporting its use in the adolescent sample.

#### Short video addiction

SVA was measured using the SVA Scale developed by Mao Zheng and Jiang Yongzhi et al. [38], which has been validated for use in adolescent populations [39]. The scale consists of 13 items across three dimensions: cognitive-behavioral changes, physiological impairment, and social entanglement. Each item is rated on a 5-point Likert scale ranging from 1 (strongly disagree) to 5 (strongly agree). Higher total scores indicate a greater level of problematic short video use or addiction. In the present study, the scale demonstrated excellent internal consistency (Cronbach’s α = 0.864).

### Covariates

The following demographic variables were included as covariates in the present study: gender, educational stage (primary, junior high, or senior high school), type of residence (urban vs. rural), only-child status, boarding status (boarding vs. day student), and left-behind status. These variables were controlled for in the analyses to account for their potential influence on the primary relationships under investigation.

### Statistical analysis

Descriptive statistics and Pearson correlation analyses were first carried out using SPSS version 23.0 to examine the basic characteristics and associations among the variables. Subsequently, a chain mediation framework was applied through the PROCESS macro for SPSS (Model 6), with PE as the predictor, SVA as the outcome variable, and DPE and PR as sequential mediators. To evaluate the significance of the mediation outcomes, a bias-corrected bootstrap procedure with 5,000 resamples was employed to produce 95% confidence intervals (95% CI) for each indirect effect. Mediating influences were regarded as significant if the corresponding confidence intervals did not include zero. The threshold of significance was set at α = 0.05.

#### Ethical approval and informed consent

This study was approved in advance by the Science and Technology Ethical Committee of Shihezi University (Approval No. KJ2024-407-02). As the participants were primary and secondary school students, some of whom were under 16 years of age, the research team clearly explained the study objectives, procedures, and principles of anonymity and confidentiality to all students and their parents or legal guardians prior to the survey. For participants under 16, written informed consent was obtained from their parents or guardians, and assent from the students themselves was also required before participation. All questionnaires were administered in paper form and were distributed and collected collectively in classrooms. Participation was entirely voluntary, and students were free to withdraw at any stage without any negative consequences. To ensure data quality, only fully completed questionnaires were included in the analysis. The study was organized and implemented by the institutions of the first and corresponding authors, under the supervision and ethical oversight of the Science and Technology Ethical Committee of Shihezi University. Therefore, the study fully complies with academic ethical standards.

## Results

### Test for common method bias

To assess common method variance, Harman’s single-factor test was applied. An unrotated exploratory factor analysis yielded three factors with eigenvalues greater than 1. The first factor accounted for 23.80% of the variance, which is well below the 40% threshold [40]. Thus, common method variance was not a serious concern in this study.

### Correlation analysis

Descriptive statistics and pearson correlation coefficients are presented in Tables 1 and 2. PE showed a significant negative correlation with both DPE (*r* = − 0.260, *P* < 0.001) and SVA (*r* = − 0.269, *P* < 0.001), and a significant positive correlation with PR (*r* = 0.229, *P* < 0.001). Additionally, PR was negatively correlated with both SVA (*r* = − 0.186, *P* < 0.001) and DPE (*r* = − 0.174, *P* < 0.001). A significant positive correlation was also found between DPE and SVA (*r* = 0.345, *P* < 0.001). These results suggest the potential mediating roles of DPE and resilience in the relationship between physical activity and problematic short video use.

**Table 1:** Descriptive statistics of demographic characteristics (*N* = 1002)

Variable	Category	*N* (%)
Gender	Boys	476(47.5%)
	Girls	526(52.5%)
Grade	Primary school	35(3.5%)
	Junior high school	394(39.3%)
	Senior high school	573(57.2%)
Place of residence	Rural	283(28.2%)
	Urban	719(71.8%)
Only child	Yes	223(22.3%)
	No	779(77.7%)
Boarding status	Boarders	320(31.9%)
	Day students	682(68.1%)
Left-behind child	Yes	499(49.8%)
	No	503(50.2%)
Age	Mean ± SD	14.78 ± 1.589

**Table 2:** Correlation analysis

Variables	1	2	3	4
1 PE	-			
2 PR	0.229^***^	-		
3 DPE	−0.260 ^***^	−0.174 ^***^	-	
4 SVA	−0.269 ^***^	−0.186 ^***^	0.345^***^	-

***: *P* < 0.001

### Mediation model analysis

**Fig. 2:**
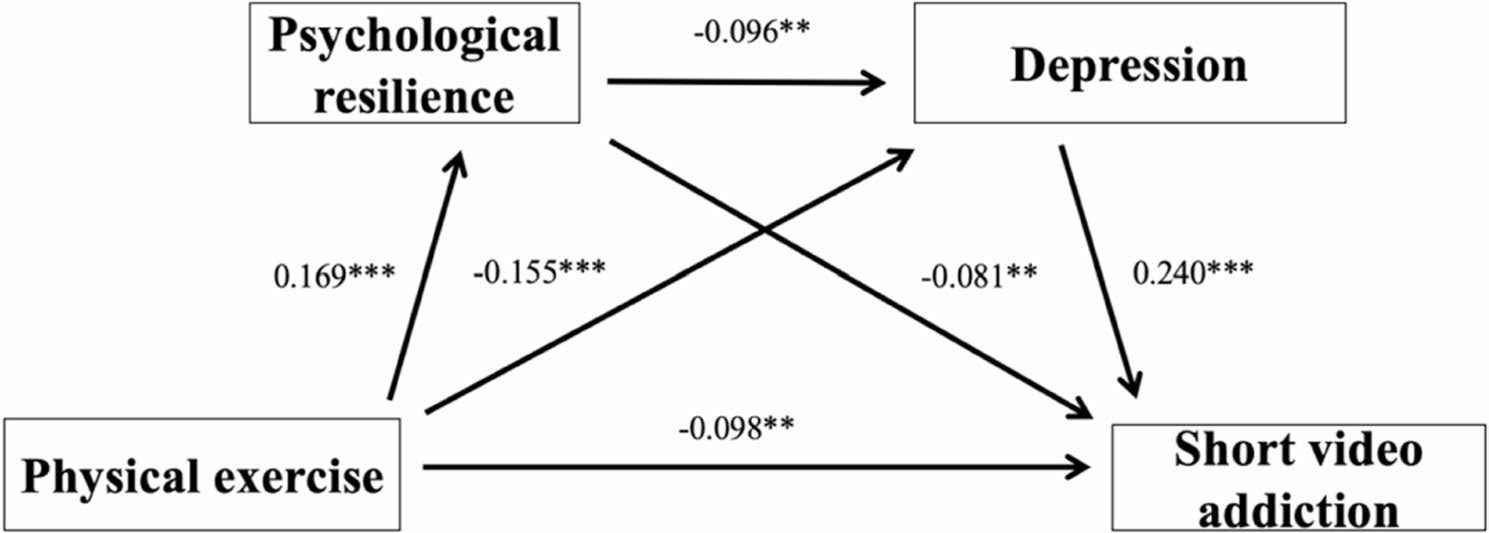
Chain mediation model

After controlling for demographic variables, the results presented in Table 3; Fig. 2 indicate that PE significantly and negatively predicts SVA (*β* = −0.153, *P* < 0.001). This predictive effect remained statistically significant even after including the mediating variables in the model (*β* = −0.098, *P* < 0.01). Moreover, PE was found to positively predict PR (*β* = 0.169, *P* < 0.001) and negatively predict DPE (*β* = −0.155, *P* < 0.001). In terms of the mediating variables, DPE showed a significant positive association with SVA (*β* = 0.240, *P* < 0.001), while PR negatively predicted SVA (*β* = −0.081, *P* < 0.01). These results support the presence of a chain mediation effect in which both PR and DPE mediate the relationship between PE and SVA. Specifically, the indirect effect via the sequential path from PE to resilience, then to DPE, and finally to SVA was statistically significant (*β* = −0.096, *P* < 0.01). The relative contribution of each mediation path is detailed in Table 4.

**Table 3:** Mediation model test

Outcome variables	Predictor variables	β	SE	t	*R*²	F
SVA	PE	−0.153	0.032	−4.816^***^	0.161	23.854^***^
PR	PE	0.169	0.033	5.079^***^	0.077	10.413^***^
DPE	PE	−0.155	0.033	−4.707^***^	0.119	14.864^***^
	PR	−0.096	0.031	−3.080^**^		
SVA	PE	−0.098	0.031	−3.127^**^	0.222	28.285^***^
	PR	−0.081	0.029	−2.770^**^		
	DPE	0.240	0.030	8.047^***^		

**: *p* < 0.01, ***: *p* < 0.001

**Table 4:** Mediation model path analysis

Path	Effect size	SE	Bootstrap 95% CI	Proportion of mediating effect
Total effect	−0.153	0.032	−0.215, −0.091	
Direct effect	−0.098	0.031	−0.159, −0.036	
Total indirect effect	−0.055	0.011	−0.077, −0.035	35.95%
PE →PR → SVA	−0.014	0.006	−0.026, −0.004	9.15%
PE → DPE →SVA	−0.037	0.009	−0.057, −0.020	24.18%
PE →PR → DPE → SVA	−0.004	0.002	−0.008, −0.001	2.61%

## Discussion

This study employed a cross-sectional design to investigate the connections among PE, PR, DPE, and SVA in adolescents. A key objective was to assess the mediating role of PR and DPE in these associations. The findings showed that PE affected SVA both directly and indirectly via its influence on PR and DPE. Specifically, PR and DPE functioned as partial intervening variables in the link between PE and SVA. Consistent with the study’s propositions, PE was negatively associated with both DPE and SVA but positively correlated with PR. Additionally, PR was positively related to PE, and DPE was positively related to SVA. These results highlight the multifaceted mechanisms through which PE may buffer against problematic digital media use among adolescents, emphasizing the significance of both emotional and PR factors in this dynamic.

This study revealed a significant negative association between PE and SVA among adolescents, which is consistent with previous research findings [14]. One possible explanation is that regular physical exercise provides individuals with a healthier source of emotional regulation and reward, helping adolescents cope with stress and boredom in a more positive way [41]. Compared with relying on short videos for instant gratification, the vitality, sense of achievement, and social connectedness brought about by exercise contribute to a more lasting sense of psychological fulfillment, thereby reducing dependence on short videos [42]. In addition, a 2025 systematic review and meta-analysis of randomized controlled trials (including 19 studies) revealed that exercise interventions significantly reduced the incidence of addictive behaviors among adolescents [43]. This evidence further supports the findings and Hypothesis 1 of the present study—namely, that physical exercise not only plays a positive role in preventing short video addiction but also promotes the overall physical and mental health of adolescents. In summary, physical exercise may serve as a potential protective factor against the risk of short video addiction [44, 45]. Schools, families, and communities should work together to create a positive exercise environment and support system, encouraging adolescents to participate regularly in physical activity to mitigate the negative impact of short video addiction and promote their psychological well-being and adaptive development [46].

This study revealed that PR acted as a partial mediator between PE and SVA, which is consistent with prior findings on behavioral addictions [47]. Regular physical exercise can enhance individuals’ coping ability and emotional regulation, thereby strengthening psychological resilience [48]. The sense of achievement, self-efficacy, and positive emotional experience gained through exercise help individuals maintain psychological stability and adaptive responses when facing stress or setbacks. In addition, physical activity provides adolescents with opportunities for social interaction and emotional support, and such positive social connections may reduce their tendencies to seek emotional gratification or escape from reality through short videos [49]. Adolescence represents a critical developmental stage during which emotional regulation and self-control are still maturing [50]. Within this context, PR serves as a protective factor, enabling adolescents to more effectively manage life stressors and negative emotions, which in turn decreases their susceptibility to the addictive use of short video platforms [51]. By fostering resilience, PE not only increases adolescents’ ability to adapt to environmental and emotional challenges but also diminishes their motivational drive for emotional escape or gratification via short video consumption [15]. These findings support Hypothesis 2 of the present study, suggesting that enhancing PR through PE offers a promising pathway to reducing SVA among adolescents. This provides both theoretical justification and practical implications for the development of targeted interventions aimed at addressing behavioral addictions among young people.

This study revealed that DPE partially mediated the relationship between PE and SVA, which is consistent with findings from prior related research [52]. Adolescence is a critical stage of transition from childhood to adulthood, during which individuals experience profound biological, psychological, and social changes [1]. Emotional regulation abilities are not yet fully developed during adolescence, leading to greater emotional volatility and a greater likelihood of experiencing depressive emotions [53]. When individuals lack effective emotional regulation strategies, they may turn to media such as short videos for emotional comfort or distraction, which can lead to addictive tendencies [54]. Physical exercise is widely recognized as a safe and effective “antidepressant” approach that promotes emotional regulation and psychological well-being by enhancing positive emotions, self-efficacy, and social connectedness [55, 56]. Previous research has shown that regular exercise helps improve mood, alleviate depressive symptoms, and consequently reduce the likelihood of relying on maladaptive coping strategies such as excessive short video use [57, 58]. Therefore, this study confirmed Hypothesis 3: depression plays a partial mediating role between physical exercise and short video addiction. Physical exercise not only directly alleviates depressive emotions but also indirectly reduces the risk of short video addiction by improving emotional regulation and social adaptability [13, 20]. In this process, physical exercise may serve as an important regulatory and protective factor, helping adolescents break this negative cycle and promoting their psychological balance and overall well-being.

This study revealed a significant negative correlation between PR and depressive symptoms, which is consistent with previous research findings [31]. These results support Hypothesis 4, suggesting that PR and DPE jointly serve as a chain mediating mechanism in the relationship between PE and SVA among adolescents. Specifically, PE enhances PR, which in turn alleviates depressive symptoms, ultimately leading to a reduction in SVA. This pathway is aligned with both ecological systems theory and the dynamic model of resilience [30], which posit that PR is not only shaped by environmental factors but that it also plays a pivotal role in an individual’s capacity to manage stress and regulate emotions. Longitudinal studies have further demonstrated that regular physical exercise can foster positive emotional experiences, strengthen adolescents’ sense of self-efficacy and social belonging, and thus enhance their PR [59]. Adolescents with higher levels of resilience are more likely to adopt adaptive emotional regulation strategies and are better equipped to cope with academic stress, interpersonal conflicts, and other adverse life events. As a result, they exhibit reduced levels of DPE and a diminished tendency to use short videos as a means of emotional escape or comfort [60]. In this context, PE indirectly mitigates SVA by reinforcing PR and lowering depressive symptoms, thereby highlighting its psychosocial value in promoting adolescent mental health.

Grounded in ecological systems theory and the dynamic model of resilience, this study developed a chain mediation model to systematically elucidate the psychological mechanisms through which PE influences SVA among adolescents. By integrating emotional regulation and PR into a unified framework, this study advances the theoretical understanding in the field of behavioral addiction. The findings demonstrate that PE is not only negatively associated with SVA but also indirectly affects it through enhancing PR and alleviating depressive symptoms. These results offer a more comprehensive sociopsychological perspective on adolescent digital addiction, providing empirical evidence that complements existing theories. From a practical standpoint, the study proposes viable implications for mental health education and addiction intervention in educational settings. It underscores the importance of a collaborative effort among educators, families, and communities to foster environments that support emotional regulation and psychological development. Encouraging adolescents to engage in regular PE can increase their psychological resources for coping with stress, thereby reducing their likelihood of resorting to maladaptive strategies such as excessive short video use in response to depressive emotions. Methodologically, this study contributes to the literature by employing a structurally integrated chain mediation model, innovatively examining the synergistic role of PR and DPE in the relationship between PE and SVA. This approach offers both theoretical and methodological innovations. Future research could further explore additional risk factors for SVA, such as adverse childhood experiences, negative interpersonal relationships, heightened emotional reactivity, and deficits in emotional regulation. Simultaneously, incorporating protective factors such as social support, positive affect, and exercise motivation may help construct more comprehensive interactional models. Longitudinal or experimental studies are also recommended to examine the causal pathways and dynamic processes underlying these relationships. Moreover, future work should investigate outcome variables influenced by SVA, including sleep quality, academic performance, and self-identity, while considering the moderating roles of demographic factors such as gender, grade level, and family background. These avenues for research can provide a stronger theoretical foundation and practical guidance for targeted interventions in adolescent populations.

Although this study revealed the psychological mechanisms through which physical exercise influences short video addiction via resilience and depression, several aspects warrant further improvement. First, the study employed convenience sampling, with participants primarily drawn from adolescents in certain regions, which may limit the representativeness of the findings. Future research could adopt stratified random sampling to increase the generalizability and external validity of the conclusions. Second, as the study used a cross-sectional design, causal relationships among variables cannot be directly inferred. Longitudinal or experimental intervention studies are recommended to further validate the dynamic causal pathways linking physical exercise and short video addiction. In addition, at the measurement level, both physical exercise and resilience were assessed using brief scales. Although these instruments demonstrated acceptable reliability, their limited dimensional coverage may have constrained the precision of construct interpretation. Future studies could employ more comprehensive and standardized measurement tools or utilize multi-indicator modeling to improve measurement stability and better capture the underlying psychological mechanisms. Overall, this study provides new empirical evidence and a theoretical framework for understanding the psychological processes linking physical exercise to short video addiction among adolescents. Despite its limitations, these constraints do not undermine the theoretical or practical significance of the findings; rather, they highlight valuable directions for future longitudinal and intervention-based research.

## Conclusion

Despite the valuable insights offered by this study into the mechanisms by which physical exercise affects short video addiction via PR and DEP, a few limitations need to be noted. First, the research adopted a convenience sampling method, which could restrict the generalizability of the results because of potential sample bias. Subsequent investigations should use stratified randomized sampling approaches to improve the representativeness of participants and strengthen the external applicability of the conclusions. Second, the cross-sectional approach of the research prevents the establishment of cause-and-effect links among the variables. To better clarify the causal mechanisms connecting PE, psychological factors, and short video addiction, future work should employ longitudinal or experimental designs. Third, regarding measurement limitations, the construct of PE was assessed using only three items, whereas PR was measured with just two items, resulting in relatively low reliability. This may compromise the stability of the measurements and the accuracy of the interpretations. Future research should consider utilizing well-validated, standardized scales with stronger psychometric properties or adopting multi-indicator approaches to improve measurement quality and more accurately capture the true relationships among variables.

## Data Availability

The datasets generated and/or analysed during the current study are not publicly available due [our experimental team’s policy] but are available from the corresponding author on reasonable request.
